# The Principles and Practice of Obstetrics

**Published:** 1861-12

**Authors:** 


The Principles and Practice of Obstetrics. By Gunning S. Bedford, A. M., M. D., Professor of Obstetrics, Diseases of Women and Children, and Clinical Obstetrics, in the University of New York; Author of " Clinical Lectures on the Diseases of Women and Children." One vol., pp. 731. S. S. & W. Wood, New York; for sale at S. C. Griggs, Chicago.

Dr. Bedford has presented the profession with a first rate work, in the book titled as above, and we are deceived, if we be not universally sustained in this opinion. Chaste, simple, yet attractive in style, full and yet concise, it will be, to the American practitioner, an interesting, as well as profitable library companion. Although given in the form of lectures,—of which there are forty-six, the arrangement and order of the subjects are natural and appropriate, enabling the author to include, in a small compass, a large amount of well digested matter. In four lithographic plates, he endeavors to give us a better idea of the appearance of the breasts, which indicate pregnancy, than can be conveyed by means of description. They are very appropriate, and form a prominent feature in the book. The Messrs. Wood have performed their part admirably well. The printing is clear and beautiful, and on excellent paper. The binding of the copy sent to us, is the only objectionable feature; it is not so substantial as a book, that will be used so much, requires, in our estimation. As an American work, we are proud of it; and would cordially recommend those in need of a text-book on obstetrics, either as an office or college companion, to buy it, believing they can do no better.

